# Discover Protein Complexes in Protein-Protein Interaction Networks Using Parametric Local Modularity

**DOI:** 10.1186/1471-2105-11-521

**Published:** 2010-10-19

**Authors:** Jongkwang Kim, Kai Tan

**Affiliations:** 1 Department of Internal Medicine, The University of Iowa, 2294 CBRB, 285 Newton Road, Iowa City, IA 52242, USA; 2 Department of Biomedical Engineering, The University of Iowa, 1402 Seamans Center, Iowa City, IA 52242, USA

## Abstract

**Background:**

Recent advances in proteomic technologies have enabled us to create detailed protein-protein interaction maps in multiple species and in both normal and diseased cells. As the size of the interaction dataset increases, powerful computational methods are required in order to effectively distil network models from large-scale interactome data.

**Results:**

We present an algorithm, miPALM (Module Inference by Parametric Local Modularity), to infer protein complexes in a protein-protein interaction network. The algorithm uses a novel graph theoretic measure, parametric local modularity, to identify highly connected sub-networks as candidate protein complexes. Using gold standard sets of protein complexes and protein function and localization annotations, we show our algorithm achieved an overall improvement over previous algorithms in terms of precision, recall, and biological relevance of the predicted complexes. We applied our algorithm to predict and characterize a set of 138 novel protein complexes in *S. cerevisiae*.

**Conclusions:**

miPALM is a novel algorithm for detecting protein complexes from large protein-protein interaction networks with improved accuracy than previous methods. The software is implemented in Matlab and is freely available at http://www.medicine.uiowa.edu/Labs/tan/software.html.

## Background

Protein complexes carry out the majority of biological processes within a cell. Correctly identifying protein complexes in an organism is useful for deciphering the molecular mechanisms underlying many cellular functions. Recent advances in proteomics technologies such as two-hybrid system and mass spectrometry has allowed enormous amount of data on protein-protein interactions (PPI) to be released into the public domain [1]. As the amount of global high throughput protein interaction data keeps increasing, methods for accurately identifying protein complexes from such data become a bottleneck for further analysis of the resulting interactome.

There is a large body of research on computational methods for *de novo *protein complex detection in PPI networks. These methods can be roughly divided into three categories. Methods in the first group define explicit complex criterion such as dense connectivity within a complex. A heuristic search strategy is then employed to identify complexes [2-4]. In contrast, the second group of methods also define a complex criterion but use complete enumeration to find all complexes that satisfy the criterion [5-7]. Instead of using local search strategy, the third group of methods are based on global graph partitioning techniques [8-11]. For instance, maximization of the modularity (*Q*) measure proposed by Newman and Girvan [12] has been successfully applied to PPI networks [11]. However, the global modularity measure has an inherent resolution limit for detecting small sub-networks [13], such as protein complexes whose median size is fewer than 10 proteins per complex. The reason for this resolution limit is that global modularity uses the entire network to compute the expected connectivity within a set of proteins, which may not be an appropriate measure of the background around protein complexes. Muff *et al*. [9] introduced a local version of the modularity measure (*LQ*) by only considering the immediate neighbors of a complex instead of the entire network. Applying it to the PPI network of *E. coli*, they showed that *LQ *was better at identifying small but biologically meaningful protein complexes.

*Q *and *LQ *represent two extremes of the neighborhood measure used to estimate background connectivity in a random network. Neither may be optimal for a given PPI network. In this study, we introduce a tunable parameter into the original formulation of modularity to help determine the optimal neighborhood size in calculating expected connectivity of a set of proteins. Another drawback of the previous *LQ *approach is that the computationally expensive optimization technique, simulated annealing, was used to maximize *LQ*, which is not feasible for large PPI networks such as yeast or human networks although it was proven useful for the smaller *E. coli *PPI network.

In this paper we introduce a novel algorithm to infer protein complexes by combining a parametric local modularity measure and a greedy search strategy. We evaluate our approach on the yeast PPI networks using two reference sets of protein complexes and additional functional annotations of yeast proteins. Compared to four existing methods, our algorithm achieves a significantly performance improvement in terms of F-measure and biological relevance of predicted complexes. By applying our method to two large-scale PPI networks, we predict a set of 138 novel protein complexes in the baker's yeast *S. cerevisiae *that warrant future experimental characterization.

## Results

### Local Modularity with Coarseness Parameter Improves Complex Prediction

**Figure 1:**
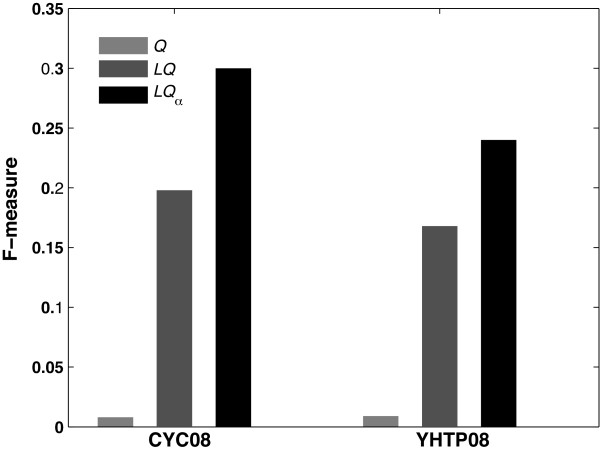
**Performance comparison of three modularity measures**. The yeast DIP full network was used as input. Optimal coarseness parameter, *a*, was optimized on three known complex sets separately.

Previously, global (*Q*) [11] and local modularity (*LQ*) [9] have been proposed as a measure to detect protein complexes in large PPI networks. However, both measures have their drawbacks. The global modularity measure has an inherent resolution limit for small sub-networks such as protein complexes [13]. The local modularity measure only considers first neighbors of a sub-network, which might not provide enough information for estimating the true background connectivity pattern of a random network. In this paper, we propose a new local modularity measure, *LQa *(parametric local modularity with the coarseness parameter *α*) for inferring protein complexes in large PPI networks. To compare how effective the three measures are to detect protein complexes, we first implemented three complex detection algorithms using a common greedy search strategy and each of the three modularity measures as the scoring function. We used the yeast full PPI network from the DIP database [14] and two sets of gold standard protein complex annotations (see Methods). As shown in Figure 1, *LQa *performed the best in terms of F-measure when evaluated using both gold standard sets. Note that *Q *and *LQ *have no coarseness parameter to set and the sets of predicted complexes are the same for the two sets of known annotations. For *LQa *we set the coarseness parameter to yield the best F-measure for each set of known complexes.

The number and average size of the predicted complexes are listed in Table 1. As expected, *Q *found a very small number of complexes with a large number of members, which caused a low recall rate and F-measure. *LQ *further resolved those large sub-networks into a number of smaller ones. However, the average size of the predicted complexes (37.5) was still much larger than the average size of known complexes (< 10). In contrast, *LQa *found a reasonable number of complexes in the same size range as the known complexes.

**Table 1:** Number and average size (arithmetic mean, in parenthesis) of predicted complexes using three different modularity measures and the DIP PPI network as input.

Complex Annotation	Modularity Measure		
	*Q*	*LQ*	*LQa*
CYC08: 236 (6.7)	27 (1877.6)	542 (37.5)	269 (4.7)
YHTP08: 207 (8.2)			262 (4.6)

*Q*, global modularity defined by Newman and Girvan [12]; *LQ*, first-neighbor local modularity defined by Muff *et al*. [9]; *LQa*, parametric local modularity defined in this study.

### Putting All Together: the miPALM Algorithm

We introduce a novel algorithm, miPALM (module inference by Parametric Local Modularity), for inferring protein complexes from large-scale protein interactome data. The input to miPALM consists of an un-weighted PPI graph and two parameters, *α *and *δ*. The algorithm has three major steps. Algorithmic details of each step and the corresponding pseudo-code are described in the Methods section. We briefly describe the major steps of the algorithm here. First, from the input PPI network, miPALM identifies a set of triangle seeds using topological overlap measure. A pair of nodes in a network has high topological overlap if they are both strongly connected to the same group of nodes (see Methods). Therefore, the use of topological overlap measure serves to exclude spurious or isolated connections in the network. Second, from each seed, the algorithm uses a greedy search to expand it into candidate complex(es). Local modularity is used as a scoring function to assess the quality of a candidate complex. The parameter *α *is used to control the background neighborhood size around a candidate complex. Finally, a filtering step is performed on the set of candidate complexes based on their density scores which is controlled by the parameter *δ*. The complete algorithm for complex prediction is shown in ***Algorithm 4***.

### Performance Comparison with Existing Methods

Next, we compare the performance of our algorithm with four representative algorithms for protein complex prediction, MCODE [2], MCL [10], COACH [15], and DME [7]. MCODE relies on the concept of K-core (a sub-graph in which all nodes have a degree at least k) and greedy search. MCL is a global graph partitioning algorithm that works by simulating stochastic flows in a graph. COACH is conceptually similar to MCODE. It first identifies the core of a candidate complex (maximal set of connected vertices whose degrees are greater than the network average) and then expand the core by including additional nodes if more than 50% of their edges are shared with the core. DME detects all node subsets that satisfy a user-defined minimum density threshold in a greedy fashion. Of the five algorithms, MCL cannot detect overlapping complexes whereas MCODE, COACH, DME, and miPALM can. Additionally, MCL is a global graph partitioning method whereas the other four are based on seeding and local search.

We tested the performance of all five methods using two sets of known complexes in the baker's yeast, *S. cerevisiae*. CYC08 is a set of protein complexes manually curated from published small-scale studies [16]. Since most small-scale studies tend to be biased towards complexes involved in a limited number of cellular processes, to complement this set, we also used the YHTP08 set of protein complexes [16]. It was constructed by analyzing two recent and most comprehensive genome-wide protein complex screens based on affinity purification coupled with mass spectrometry experiment [17,18].

**Figure 2:**
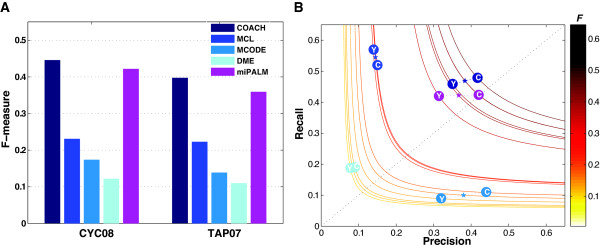
**Performance comparison of five complex detection algorithms**. **A) **F-measure of the five algorithms with their best parameters optimized to two sets of known complexes CYC08 and YHTP08 using the DIP network as input. **B) **Precision and recall of the methods. Circles, F-measures measured against CYC08 (C) or YHTP08 (Y). Lines, F-measure contours. Two different points on the precision-recall plane can have the same F-measure values if they lie on the same F-measure iso-line. The average F-measure of an algorithm, over the two gold standard sets, is indicated with a star.

**Figure 3:**
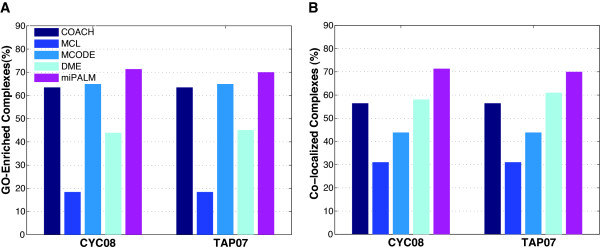
**Biological relevance of predicted complexes by five algorithms with optimized parameters and the DIP PPI network as input**. **A) **Percentage of complexes enriched for at least one GO term. **B) **Percentage of complexes whose members are co-localized in the same sub-cellular compartment.

For performance comparison we determined the optimal parameters for each algorithm to achieve the highest F-measure, given a gold standard set (see Methods). The comparison results are presented in Figures 2 and 3 and Table 2. For each method, we report the precision, recall, and F-measure. As can be seen in Figure 2A, both COACH and miPALM achieved a much higher F-measure compared to the other three methods. The average F-measure was 0.42, 0.39, 0.23, 0.16, and 0.12 for COACH, miPALM, MCL, MCODE, and DME, respectively.

**Table 2:** Statistics of predicted complexes by five algorithms with the best parameters optimized on CYC08 and YHTP08 sets and the DIP PPI network as input.

Algorithm	Gold Standard Sets	
(optimized parameters)	CYC08 (236/6.7)	YHTP08 (207/8.2)
COACH	271/113/7.3	271/95/7.3
(affinity threshold)	(0.1)	(0.1)

MCODE	57/25/12.9	57/18/12.9
*(VWP)*	*(0.2)*	*(0.2)*

MCL	830/123/5.9	830/115/5.9
*(inflation)*	*(1.75)*	*(1.75)*

DME	487/44/25.1	503/40/24.7
*(density threshold)*	*(0.97)*	*(0.96)*

miPALM	238/100/7.0	277/88/7.0
*(α, δ)*	*(0.364, 2.40)*	*(0.374, 2.33)*

Total number and average size of gold standard sets are shown in parenthesis. The series of three numbers for each set of predictions are total number of predictions, number of predicted complexes that overlap with known complexes, and average size of predicted complexes. Optimized parameters for each algorithm are shown in parenthesis following prediction numbers.

Figure 2B shows a breakdown of the F-measure into precision and recall for all five methods. On average, MCL achieved the highest recall mainly due to its large number of predictions. On the other hand, MCODE achieved the highest precision because it tends to identify a subset of known complexes with higher overlap than other methods. However, the overall accuracy of both methods (as measured by the F-measure) was lower than those of COACH and miPALM because MCL had a much lower precision and MCODE had a much lower recall. In other words, the higher F-measure achieved by COACH and miPALM is due to a balanced increase in both their recall and precision.

Although F-measure is a popular metric for evaluating the performance of a complex predictor, it is not the only one. Biological relevance is also an important indicator of the quality of predicted complexes. Accordingly, we next conducted GO term enrichment and co-localization analyses to determine the biological relevance of the predicted complexes. Genome-wide protein localization data has been reported for Baker's yeast using fluorescent imaging [19]. For each predicted complex, we calculated a log-odds score that measures the extent to which members of the complex co-localize to the same sub-cellular compartments (see Methods). Compared to the F-measure that relies on an incomplete gold standard set, both GO term and co-localization annotations used here are more comprehensive and thus complementary to the F-measure.

At a *p-value *of 0.05, our set of predictions had the highest fractions of complexes with enriched functional categories (Figure 3A). Compared to the second best performer (MCODE), the average increase in the fraction of enriched complexes was 8.9% across the two gold standard sets of complexes. For complex member co-localization, our predictions had an 18.8% average increase compared to the second best performer, DME (Figure 3B).

Taken together, our benchmarking analyses demonstrated that miPALM achieved the second highest F-measure (3% lower than COACH) when evaluated using known complexes. On the other hand, miPALM outperforms all other algorithms by a large margin (8.9% and 18.8%) when evaluated using functional annotations of complex members.

### Novel Complex Predictions Using Large Yeast PPI Networks

Next, we applied miPALM to discover novel protein complexes in two large-scale yeast PPI networks based on interactions obtained from the BioGRID database [20]. The first network consists of all yeast interactions in the BioGRID database. The majority of interactions are derived from high throughput experiments. The second network consists of high-confidence interactions derived by filtering the BioGRID interactions based on their lines of supporting evidence [21]. For brevity's sake, these two networks are termed BioGRID and HC networks in this paper. The BioGRID network contains 5591 proteins and 51880 physical interactions and the HC network contains 2228 proteins and 6209 physical interactions. By studying two networks with different amount of noise, we can assess the robustness of our method on noisy data.

To predict complexes, we set the coarseness parameter *α *to be 0.364 that gave the highest F-measure as described in the performance comparison section.

**Figure 4:**
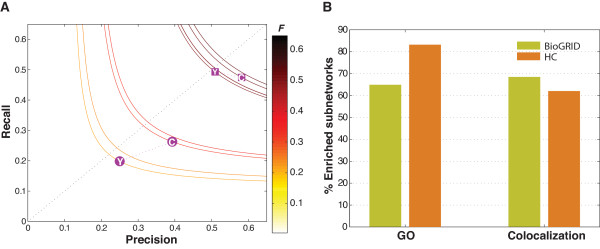
**F-measure and biological relevance of predicted complexes using two large-scale PPI networks as inputs**. **A) **F-measures. Circles, F-measures measured against CYC08 (C) or YHTP08 (Y) using the BioGRID network as input. Squares, F-measures measured against CYC08 (C) or YHTP08 (Y) using the HC network as input. **B) **Fraction of predicted complexes enriched for GO term and colocalization.

**Figure 5:**
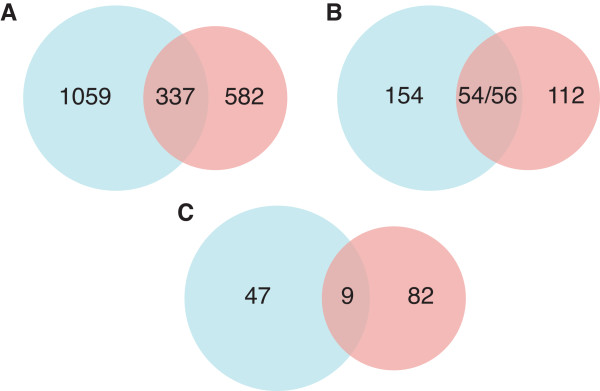
**Overlap between miPALM predicted complexes using two large-scale PPI networks as inputs**. **A) **Overlap between protein members in the predicted complexes. **B) **Overlap between complexes. **C) **Overlap between novel complexes. Blue, predicted complexes using HC PPI network; Red, predicted complexes using BioGRID PPI network.

In total, miPALM predicted 168 and 208 protein complexes from the BioGRID and HC network, respectively. The respective F-measures for the two sets of predictions are 0.31 and 0.52 (Figure 4A). As expected, predictions using HC network has a higher F-measure due to the higher quality of the input data. Nevertheless, as shown in Figure 5, the two sets of complexes overlap by 33.3% (56/168). To assess the significance of the overlap, we also used the other four methods in the benchmarking study to predict complexes in the BioGRID and HC networks. We used the same optimized parameters for each method as described in the performance comparison section. The two sets of complexes predicted by COACH had the highest overlap of 43.3%. The average overlap for the four methods was 26.6%. As an additional check, we considered miPALM predictions using the DIP networks as input. The average overlap between the three sets of predictions is 38.3% (Figures S6, S7 in Additional file 1). Taken together, the high level of overlap between miPALM predictions suggests that it is fairly robust against noisy data.

After merging overlapped complexes, we ended up with 322 predicted complexes from the two networks. Two hundred thirty two of these complexes (72.5%) are enriched for at least one GO term (Table 3), suggesting many of them are true protein complexes. Examined separately, 109 (64.9%) BioGRID and 173 (83.2%) HC predictions are enriched for at least one GO term, respectively (Figure 4B).

**Table 3:** Supporting evidence for novel complexes predicted by miPALM compared to gold standard sets of known complexes.

	CYC08 (%)	YHTP08 (%)	miPALM All (%)	miPALM Novel (%)
GO	76.7	56.5	72.5	61.6

Colocalization	25.9	43.0	65.0	68.8

BioGRID and HC PPI networks were used as the input. GO, gene ontology.

To further corroborate our predictions, we next used a genome-wide protein localization data set to examine if members of our predicted complexes tend to co-localize in the same sub-cellular compartments. For each of our predicted complex, we calculated a co-localization log-odds score that compares the member co-localization probability of a predicted complex to the probability of the same number of random proteins in the PPI network (See Methods). For the set of 320 predicted complexes, 208 (65.0%) are enriched for at least one sub-cellular compartments (Table 3). Examined separately, 115 (68.5%) BioGRID and 123 (62.0%) HC predictions are enriched for at least one sub-cellular compartment, respectively (Figure 4B).

To identify new complexes in our prediction, we used the union of CYC08 and YHTP08 as the set of known complexes. After filtering those complexes matching any of the known complexes, we were left with 138 novel protein complexes. To evaluate the quality of these novel protein complexes, we computed the fraction of complexes that have enriched GO functional terms or are co-localized to the same sub-cellular compartments. Eight five (61.6%) of the novel complexes were enriched for at least one GO terms and 95 (68.8%) complexes were enriched for at least one sub-cellular compartments (Table 3). The fraction of GO term enriched complexes was comparable to known complexes. Remarkably, the fraction of co-localized complexes in our prediction was much higher than those of the two gold standard sets (Table 3). These results provide further evidence that the set of novel complexes are true protein complexes. Information about the complete set of predicted complexes with supporting evidence is reported in Additional files 1, 2, 3 and 4.

## Discussion

The global modularity measure proposed by Newman and Girvan [12] identifies clusters (sub-networks) in a network by comparing the observed fraction of edges inside a cluster to the expected fraction of edges in the cluster. In doing so, it assumes that connections between all pairs of nodes in the network are equally probable, which reflects all connectivity among all clusters. However, in many molecular interaction networks, most sub-networks are only connected locally. For instance, in metabolic networks, major pathways occur as clusters that are sparsely linked among each other [22]. The same observation can also be made on protein complexes [23].

In this study, we introduced parametric local modularity as a new measure for the quality of clusters in a network. It takes into account local cluster connectivity and overcomes global network dependency. As an analogy, the coarseness parameter functions as the resolution dial of a microscope. By changing the value of the coarseness parameter, we can adjust the size of the cluster neighborhoods when calculating the expected fraction of edges within a cluster. Since different biological networks might have distinct neighborhood connectivity, a tunable local modularity measure allow us to best estimate the local neighborhood connectivity by changing the size of the neigbhorhood under consideration.

Protein complexes are dynamic molecular entities. Depending on the cellular states, membership of a protein complex could change and different complexes could have shared members [18]. Our algorithm can detect overlapping complexes if during the seed expansion step seeds of different candidate complexes are close enough.

The F-measure used for performance evaluation is a popular approach. A drawback of F-measure is that it cannot distinguish whether a predicted complex overlap with just one or multiple known complexes and vice versa. It has been argued that predictions that overlap with fewer known complexes should be regarded as having a higher quality [24]. To further evaluate the methods using this criterion, we use the separation metric introduced by Brohee and van Helden [24] which takes into account the observation above. As shown in Figure S8 (Additional file 1), miPALM again outperforms the other methods. Therefore, it is unlikely that the performance improvement by miPALM is due to a bias in the benchmarking metrics used.

In summary, using three alternative performance measures (F-measure, Biological Relevance, Separation), our benchmarking analysis demonstrate that miPALM achieve an overal best performance among the five algorithms compared. The performance measures of the methods using three input interaction networks are summarized in Additional file 1, Tables S4, S5, S6.

The proposed algorithm can be naturally extended to handle weighted networks by using edge weights for local modularity calculation. Edge weights can be calculated based on topological features of the PPI network and domain-specific information from other omic data, such as microarray gene expression, genome-wide association study, and genome-wide sequence mutation data (e.g. cancer mutation screening). Integration of functional genomic data into miPALM will enable us to find context-dependent sub-networks that are active under specific growth conditions.

## Conclusions

Using several performance measures (F-measure, Biological Relevance, and Separation), we have demonstrated that miPALM achieved an overall improvement over previous algorithms. miPALM combines the strength of three key features, triangle seed identification using topological overlap measure, parametric local modularity as a cluster quality measure, and recursive greedy search. By including functional genomic data as edge weights, miPALM can be extended to identify context-dependent gene modules that can in turn be used to assist in network comparison and classification tasks.

## Methods

### Protein interaction and complex data

#### Protein interaction networks

Yeast protein-protein interaction data were downloaded from the DIP [14] and BioGRID [20] databases. The DIP "full" set of PPIs (including all physical interactions in the DIP database instead of a subset of high confidence interactions) were used for algorithm development and comparison. The BioGRID and high-confidence [21] sets of PPIs were used for novel protein complex prediction. After removing self-loops and multiple edges, the three networks contain 4859, 5591, and 2228 proteins and 17138, 51880, and 6209 interactions, respectively.

#### Known annotated protein complexes

Two sets of annotated protein complexes were used for performance evaluation. Pu *et al*. generated a comprehensive catalogue of 408 protein complexes manually curated from published small-scale experiments reported as of 2008 [16]. This set provides an update of the widely used gold-standard MIPS complexes. In the same study, they also generated a catalogue of 400 high-throughput complexes by a systematic analysis of all high throughput protein-protein interaction data reported as of 2008. After removing complexes with fewer than 3 members, we ended up with two reference sets of protein complexes, termed CYC08 (236 complexes) and YHTP08 (207 complexes), respectively.

### Construction of the seed set

Seeding strategy is crucial for a network searching algorithm since the search result is dependent on the starting point (e.g. a node, an edge, or a sub-network). Here we describe how to construct seeds and to rank them based on the local property of the network.

First, we weight every interaction in the PPI network. For discovering good seeds, it is important to rank within-complex edges high and between-complex edges low. We used a modified version of the topological overlap measure by Ravasz *et al*. [25] as edge weight. It is defined as following:

(1) $$O_{T}(v,w)=A_{vw}\cdot \left|\Gamma (v,w)\right|/(k_{v}+k_{w})/2$$

where |Γ(*v*, *w*)| is the number of common neighbors of node *v *and *w*, *k_v _*and *k_w _*are the degrees of node *v *and *w*, *A_vw _*= 1 if *v *and *w *have a direct link and zero otherwise.

In the original definition of *O_T _*(*v, w*), the number of shared interacting partners is normalized by dividing |Γ(*v*, *w*)| by *min*(*k_v_*, *k_w_*) instead of *(k_v _+ k_w_)*/*2*. We modified the normalization factor because it is improper to treat two proteins topologically equal if one protein has three interactors and the other has 100 interactors (e.g. hub proteins) even though these two proteins share the same three interacting partners.

Second, we enumerated all triangles in the PPI network using the enumeration algorithm described in *Algorithm 1*. All triangles in the PPI network can be located by ***Algorithm 1 ***in O(k_max_·m) time with an upper bound of O(n·m), where *k_max _*is the largest node degree in the network.

**Algorithm 1: ***TriangleEnumeration *(G)

1   **input**: Unweighted graph *G *= (*V*, *E*)

2   **output**: all triangles of *G*

3   **begin**

4    **for ***e ****∈ ****E ***do**

5         *(v, w) ← *a pair of nodes connected by *e*

6         *Γ (v, w) ← *a set of common nodes shared by *v *and *w*

7         **for ***x ****∈ *Γ***(v, w) ***do**

8            output triplet {*v, w, x*}

9         remove *e *from *G*

10   **end**

We then rank all triangles found by ***Algorithm 1 ***based on their triangle-weights obtained by averaging pair-wise edge weights.

### Local modularity as the scoring function

The total modularity *Q *of a network with *M *modules is defined as following [12]:

(2) $$Q=\sum_{s=1}^{M}Q_{s}=\sum_{s=1}^{M}\left[\frac{m_{ss}}{m}-{\left(\frac{d_{s}}{2m}\right)}^{2}\right]$$

where *m *is the total number of edges in the network, *m_ss _*is the number of intra-module edges in module *S*, and *d_s _*is the sum of the degrees of nodes in module *S*. Essentially, *Q *is the difference in the fraction of within-module edges between the observed network and a random configuration network model. This definition of modularity is global in the sense that the comparison of *m_ss_/m *with (*d_s_/2m*)^2 ^assumes equal probability of connection between any pair of nodes in the random network model.

During module search, when a node *v *and a sub-network *S *are merged, the change in global modularity can be derived, as followings,

(3) $$\Delta Q(v,\;S)=Q_{vS}-(Q_{v}+Q_{S})=\frac{1}{m}\left(m_{vS}-\frac{d_{v}d_{S}}{2m}\right)\;$$

where *Q_v _*and *Q_S _*are the modularity of *v *and *S*, respectively and *Q_vS _*is the modularity of the sub-network created by merging *v *and *S*.

In order to overcome the resolution limit of the global modularity measure, Muff *et al*. proposed the local modularity measure *LQ *[9]

(4) $$LQ=\sum_{s=1}^{M}\left[\frac{m_{ss}}{m_{s}}-{\left(\frac{d_{s}}{2m_{s}}\right)}^{2}\right]\;$$

where *m_ss _*is the number of edges within sub-network *S *and *m_s _*is the total number of edges in *S *and its *first *neighbours. *LQ *is based on the observation that in real world networks most sub-networks are only connected to a small fraction of the entire network.

Inspired by previous work, we introduce a new local modularity measure for a single subnetwork as defined below:

(5) $$LQ_{\alpha }=\frac{m_{SS}}{m}-{\left(\frac{d_{S}}{2m^{(\alpha +1)/2}}\right)}^{2},\;0\leq \alpha \leq 1$$

where the denominator of the second term in Eq. 4 is not fixed to *2m*, but varied with a parameter *α *that we call the *coarseness parameter*.

After merging *v *and *S *the change in the newly defined local modularity is then:

(6) $$\Delta LQ_{\alpha }(v,\;S)=\frac{1}{m}\left(m_{vS}-\frac{d_{v}d_{S}}{2m^{\alpha }}\right),\;0\leq \alpha \leq 1\;$$

Readers are referred to the Suppl. Methods (Additional file 1) for detailed derivation of Δ*LQ_α _*from *LQ_α_*

When *α = 1*, Δ*LQα *is equivalent to Δ*Q *in Eq. 3. Decreasing *α *leads to a smaller number of edges to be considered. For example, if *α *= 0.5, the ratio of considered edges to the total number of edges in the network (i.e. edge-coverage ratio, *r*= 2*m^α ^*/2*m *)) is *m*^-1/2^. Conversely, if we want to cover locally 50% of edges (*r = 0.5*), then *α *can be set to *1+log_m_(0.5)*. As *α *goes down to zero, the size of the detected sub-network becomes smaller and smaller because the expected fraction of within-module edges, the second term in Eq. 5, becomes larger. Suppl. Figure S1 (Additional file 1) shows the edge-coverage ratio and size of resultant detected sub-networks as a function of *α*.

### Greedy search by maximizing local modularity measure

The problem of finding a network partition with maximum global modularity is known to be NP-hard [26]. Thus, various heuristic approaches were proposed [27-32]. In particular, greedy search [31,32] based on global modularity have been studied extensively due to its single peakness [33] and fast speed for analyzing very large networks.

Our scoring function (Eq. 5) made it possible to adopt a greedy search strategy to expand a given triangle seed to a larger sub-network iteratively until the increase in local modularity becomes negative. Pseudo codes for our greedy search algorithm are shown in ***Algorithms 2 ***and ***3***. Briefly, starting with the top ranked triangle seed *{x*, *y, z}*, our greedy algorithm always merge the direct neighbor *w *of the seed that increases local modularity the most, growing the seed into a larger sub-network *S={w*, *x, y, z}*. The algorithm outputs *S *if it has no additional neighbor merging of which leads to an increase in the local modularity. This searching process (or seed expansion) is then repeated with a new seed. The time-consuming step of the greedy search algorithm is the calculation of Δ*LQ_α _*after each merging. We avoid recalculating Δ*LQ_α_*(*v*, *S'*) for all neighbours of *S'*, v*∈*N_s' _by taking advantage of the recursive relationship for Δ*LQ_α _*between before and after merging (see Suppl. Methods and Figure S3 for details, Additional file 1). The upper bound for the time complexity of our search algorithm is *O(n_s_*·*d_s_) *where *n_s _*is the number of proteins in the sub-network *S *and *d_s _*is the sum of degrees of all nodes in the sub-network *S*.

**Algorithm 2: ***RecursiveGreedySearch *(*S*, *A*, *α*)

1   **input**: triangle seed *S*, adjacency matrix *A*, and coarseness parameter *α*

2   **output**: Expanded sub-network *S^' ^*and its neighbor nodes *N_s'_*

3   **begin**

      *Ns ← *neighbor nodes of *S*

*5      *Δ*LQ_α _(*·, *S) ← *change in our local modularity for all *v *in *N_s_*

6      **if **max((Δ*LQ_α _*(·, *S*)) < 0 **then**

7         **return ***S *and *N_s_*

8      [*S'*, *N_s'_*] *← GrowSeed*(*S*, *A*, *N_s_*, *α*, Δ*LQ_α _(·, S)*)

9      **return ***S' *and *N_s'_*

10   **end**

**Algorithm 3: ***GrowSeed *(*S*, *A*, *N_s_*, *α*, Δ*LQ_α _*(·,*S*))

1   **input**: triangle seed *S*, adjacency matrix *A*, a set of neighbor nodes of *S N_s_*, coarseness parameter *α*, change in local modularity Δ*LQ_α _(v, S) *for all *v *in *N_s_*

2   **output**: Expanded sub-network *S^' ^*and its neighbor nodes *N_s'_*

3   **begin**

4      $v^{*}\leftarrow arg\underset{v}{max}\{\Delta LQ_{\alpha }(v,S)\}$

5      N_v* _← all neighbor nodes of v*

6      *S' ← *{*S*, v*}

7      $N_{s^{\prime }}\leftarrow (N_{s}-\{v^{*}\})\cup (N_{v*}-(N_{s}\cup S)),\;v\in N_{s}-\{v^{*}\}$

8      $\Delta LQ_{\alpha }(v,\;s^{\prime })\leftarrow \Delta LQ_{a}(v,\;S)+\Delta LQ_{a}(v,\;v^{*}),\;v\in N_{v*}-(N_{s}\cup S)$

9      $\Delta LQ_{a}(v,\;S^{\prime })\leftarrow -\frac{d_{v}d_{s}}{2m^{\alpha +1}}+\Delta LQ_{\alpha }(v,\;v^{*})$

10      **if **max (Δ*LQ_α _*(***·***,*S'*)) *< 0 ***then**

11         **return ***S' *and *N_s'_*

12      $\left[S',N_{s'}\right]\leftarrow GrowSeed(S',\;A,\;\alpha ,N_{s'},\;\Delta LQ_{\alpha }(\cdot ,S'))$

13   **end**

### Elimination of unpromising seeds

Unpromising seeds are those that cannot be expanded into larger sub-networks. In other words, they are triangles that have no neighbors that can cause positive change in local modularity if merged. We filtered out those triangles after seed expansion step to speed up the algorithm and reduce the number of false positives (see Figure S2 in Additional file 1).

### Complex merging

Proteins in a PPI network could belong to one or more protein complexes simultaneously. This multiple membership of proteins should be uncovered by the clustering algorithm. Complexes found by our method can be overlapped if they are within the same densely connected region in the PPI network. While revealing overlapped complexes is important for understanding their dynamics, allowing algorithm to make overlapped predictions often produce an excessive number of complexes. For example, the algorithm DME [7] predicted 14,780 complexes (minimum density threshold 0.95) on the yeast DIP full set. The majority of them are overlapped, causing low precision and poor overall performance. In this paper we merged any two complexes *S *and *T *if they have an overlap score of greater than 0.5, which is defined as |*S *⋂ *T*|/*min*(|*S*|, |*T*|).

### Complex filtering by density score

After merging complexes produced by the seed expansion step, we rank the candidate complexes by their density score *δ_s _*that is defined as the product of the connectivity and size of complex $S,\;\delta_{s}=\frac{m_{ss}}{n_{s}(n_{s}-1)/2}\cdot n_{s}$.

### The miPALM algorithm

Our algorithm takes as input an unweighted PPI network *Gn, m*={*V, E*} with *n *nodes and *m *edges and outputs a set of predicted protein complexes, *M*. The pseudo code of the algorithm is shown in ***Algorithm 4***.

**Algorithm 4: **miPALM (*G*, *α*, *δ*)

1   **Input**: Unweighted graph *Gn, m *={*V*, *E}*, *n=/V/, m=/E*|, coarseness parameter *α*, and density score threshold *δ*

3   **Output**: a set of sub-networks, *M*

4   **begin**

5      *T ← TriangleEnumeration *(*G*)

6      *t ← *choose the top ranked triad-seed in *T*

7      *T ← *delete *t *from list *T*

8      **while ***T *is not empty **do**

9         *S ← RecursiveGreedySearch *(*t*, *A*, *α*)

10         *t ← *choose the top triad-seed uncovered by the previous search

11         *T ← *delete *t *from list *T*

12         **if **the size of *S *is three **then**

13         continue

14         *S ← *refine *S *by looking around *S*

15         *M ← *{*M*, *S*}, output *S*

16      *S ← *merge sub-networks in *S*

17      **for ***S ***∈ ***M ***do**

*18         δ_s _← *get density score f *S*

19         **if ***δ_s _*<*δ ***then**

20            delete *S *from *M*

21   **end**

### Performance evaluation

We used the F-measure to evaluate the performance of complex prediction algorithms. F-measure is the harmonic mean of the two quantities, precision (Pre) and recall (Rec), 2 Pre Rec/(Pre + Rec). Precision is defined as the ratio of the number of matched sub-networks to the number of predicted sub-networks by each algorithm. Recall is the ratio of the number of matched sub-networks to the number of known complexes.

For comparison purpose, we used the complex matching criterion used in MCODE [2] to identify predicted complexes that overlap with gold standard complexes. A predicted sub-network is considered matched to a known complex if it has a matching score of 0.2 or greater. Matching score is defined as *ω = c^2^/a·b*, where *a*, *b *are the size of the sub-network and the known complex, respectively, and *c *is the number of protein members overlapped between the prediction and the known complex. We also examine the precision and recall rates at different overlap scores (see Figure S9 in Additional file 1).

### Parameter selection

Our algorithm has two parameters, *α *for determining the size of the local neighborhood of a candidate complex and *δ *for filtering candidate complexes based on their density score. For benchmarking purpose, we used the *F-measure *to determine the parameters yielding the best performance of the algorithm on three sets of known complex. Because the *δ *parameter is only used for post-search filtering, we first searched for the optimal *α *value. We varied *α *from 0 to 1 with an initial step size of 0.01. Once the range of optimal *α *value was located, we further searched for the optimal parameter value using a finer step size of 0.001 (Figure S4 in Additional file 1). After an optimal *α *was found, we determined the optimal *δ *by searching from 0 to 3.5 with a step size of 0.01. To determine the sensitivity of the algorithm to parameter changes, we determined the overlaps between predicted complexes using two *α *values differed by 0.01. As can be seen in Figure S5 (Additional file 1), our algorithm is not overly sensitive to parameter changes.

For the other four programs we compared, we tested the following parameter ranges that gave optimal *F-measure *on the three sets of known complexes. For COACH, the affinity threshold was varied from 0 to 1 with a step size of 0.01. For MCL, the inflation parameter was varied from 1.2 to 5.0 with a step size of 0.01. For DME, the density threshold parameter was varied from 0.91 to 1.0 with a step size of 0.01. For MCODE, vertex weight percentage = 0.2, haircut = TRUE, and fluff = FALSE were used. These parameters of MCODE have been optimized to produce the best results by default.

### Gene ontology term enrichment test

Yeast Gene Ontology (GO) slim terms were used to evaluate the biological relevance of predicted complexes. P-value for GO term enrichment was calculated using the hypergeometric distribution. A Bonferroni-corrected p-value of 0.05 is considered to be significant.

### Co-localization analysis

Based on fluorescence imaging, Huh *et al*. [19] classified 75% of the yeast proteome into 22 distinct sub-cellular compartments. Protein localization data was downloaded from the yeast GFP fusion localization database http://yeastgfp.yeastgenome.org. To compute a log-odds score of complex sub-cellular localization, we compared the observed number of protein pairs within a sub-network *S *that are co-localized to sub-cellular compartment *k *(*m_sk_*) to the expected number of such pairs in a random network $\bar{m_{sk}}$, defined as following,

$$\bar{m_{sk}}=\frac{n_{sk}(n_{sk}-1)}{2}\cdot p_{s}$$

and

$$p_{s}=2\cdot m_{ss}/n_{s}\left(n_{s}–1\right)$$

where *n_sk _*is the number of proteins localized in compartment *k *in sub-network *S *and *p_s _*is the connectivity for the sub-network. We consider a complex to be localized to a compartment *k *if the log-odds score $log({}^{m_{sk}}/{}_{\bar{m_{sk}}})>0$.

## Authors' contributions

JK and KT conceived and designed the study. JK performed the experiments. JK and KT analyzed the data. All authors have read and approved the final manuscript.

## Supplementary Material

Additional file 1 **Supplemental Methods and Materials**. Click here for file

Additional file 2 **Supplemental Table S1**. Click here for file

Additional file 3 **Supplemental Table S2**. Click here for file

Additional file 4 **Supplemental Table S3**. Click here for file
