# Supplementary material for: Discover Protein Complexes in Protein-Protein Interaction Networks Using Parametric Local Modularity
Source: BMC Bioinformatics. 2010 Oct 19;11:521. doi: 10.1186/1471-2105-11-521 (PMC2974752; doi:10.1186/1471-2105-11-521)
Supplement: Additional file 1 — Supplemental Methods and Materials. [file 1471-2105-11-521-S1.PDF]

Supplemental Methods and Materials

Derivation of (Eq. 6) from parametric local modularity (Eq. 5)

ΔLQ_α in (Eq. 6) is derived from LQ_α (Eq. 5)

$$LQ_\alpha(S) = \frac{m_{SS}}{m} - \left(\frac{d_S}{2m^{(\alpha+1)/2}}\right)^2, \quad 0 \le \alpha \le 1$$

$$\Delta LQ_\alpha(S,v) = LQ_\alpha(S \cup v) - [LQ_\alpha(S) + LQ_\alpha(v)]$$

$$= \left[\frac{m_{ss} + m_{sv}}{m} - \left(\frac{d_s + d_v}{2}\right)^2 \frac{1}{m^{\alpha+1}}\right] - \left[\frac{m_{ss}}{m} - \left(\frac{d_s}{2}\right)^2 \frac{1}{m^{\alpha+1}} - \left(\frac{d_v}{2}\right)^2 \frac{1}{m^{\alpha+1}}\right]$$

$$= \frac{m_{sv}}{m} - \frac{d_s d_v}{2m^{\alpha+1}}$$

$$= \frac{1}{m}\left(m_{sv} - \frac{d_s d_v}{2m^{\alpha}}\right)$$

$$= \Delta LQ_\alpha(S,v)$$

Recursive searching algorithm

In this section we describe the fast version of our greedy search algorithm for expanding seeds. Given a sub-network S (see Figure S2), the algorithm finds the best neighbor v^* that gives maximum increase in local modularity ($\Delta LQ_\alpha(v^*, S) = \max_{v \in N_s}\{\Delta LQ_\alpha(v, s)\}$) among all $v \in N_s$ where N_s is the set of neighbors of S. The time-consuming step of the search algorithm is the calculation of ΔLQ_α after each merging. We avoid recalculating $\Delta LQ_\alpha(v, S')$ for all neighbors of S', $v \in N_{S'}$ by taking advantage of the recursive relationship of ΔLQ_α between before and after merging:

$$\Delta LQ_\alpha(v, S') = \Delta LQ_\alpha(v, S) + \Delta LQ_\alpha(v, v^*) \quad \textit{for } v \in N_s - \{v^*\} \text{(Eq. 1)}$$

where $v^* = arg\, max_{v \in N_s}\{\Delta LQ_\alpha(v, S)\}$.

Figure S3 illustrates the relationship of the sub-network before merging (S) and after merging (S'). Based on this relationship we don't have to identify the set of neighbors of

S' since $N_{s'}$ and N_s share many common members, thus reducing the computing time. Derivation of Eq. 1 is shown below.

By definition,

$$\Delta LQ_{vS'} = \frac{1}{m}\left(m_{vS'} - \frac{d_v d_{s'}}{2m^{\alpha}}\right) \text{ for all } v \in N_{s'}.$$

Since $m_{vS'} = m_{vS} + A_{vv^*}$, $d_{S'} = d_s + d_{v^*}$, substituting these two terms into the equation above yields

$$\Delta LQ_{vS'} = \frac{1}{m}\left[m_{vS} + A_{vv^*} - \frac{d_v(d_S + d_{v^*})}{2m^{\alpha}}\right]$$

$$= \frac{1}{m}\left(m_{vS} - \frac{d_v d_S}{2m^{\alpha}}\right) + \frac{1}{m}\left(A_{vv^*} - \frac{d_v d_{v^*}}{2m^{\alpha}}\right)$$

$$= LQ_{vS'} + LQ_{vv^*}$$

Note that $m_{vS'} = A_{vv^*}$ for $v \in N_{v^*} - (N_s \cup S)$ since $m_{vS} = 0$.

In summary,

$$\Delta LQ_{vS'} = \begin{cases} \Delta LQ_{vS} + \Delta LQ_{vv^*} for v \in N_s - \{v^*\} \\ -\frac{d_v d_s}{2m^{\alpha+1}} + \Delta LQ_{vv^*} for v \in N_{v^*} - \{N_S \cup S\} \end{cases}$$

Algorithm for merging overlapping subnetworks

Algorithm 5: MergeSubnet(G)

Input: A list of predicted sub-networks. Each line represents a sub-network and contains a set of node IDs arranged in ascending order.
Output: A list of merged sub-networks
begin
$O_{ij} \leftarrow$ get overlap score for all pairs of sub-networks *i* and *j*
while $O_{st} \geq 0.5$
    $O_{st} \leftarrow$ find the biggest overlap score and its index *s* and *t* $(s \neq t)$
    Merge two lines into a new line, and delete line *s* and *t*
    $O_{ij} \leftarrow$ get overlap score for all pairs of sub-networks *i* and *j*
end

Figure S1. Edge coverage ratio and sub-network size as a function of the coarseness parameter, *α*.

Network is the yeast DIP full PPI network. Solid line, edge coverage ratio. Circle, relative size of the sub-network obtained by expanding from the top-ranked triangle seed. Both edge coverage ratio and sub-network size increase exponentially as *α* increases. For *α* = 0.5, only 1.58% of the total number of edges are taken into account in calculating the change of local modularity and 0.27% of nodes in the network are covered by the predicted complex.

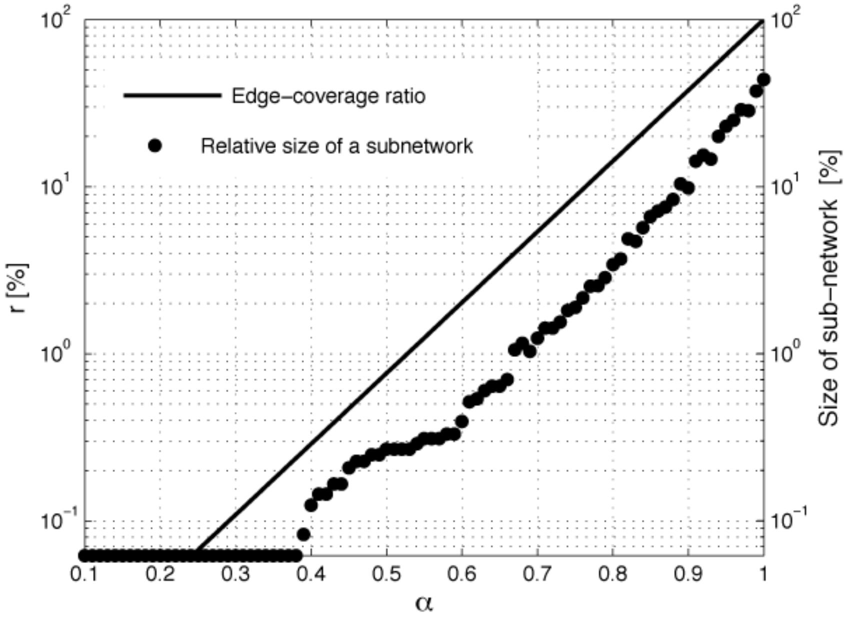

Figure S2. Performance of miPALM with and without elimination (skipping) of unpromising triangle seeds. Unpromising seeds are those that cannot be expanded into larger sub-networks. A) F-measure of the two versions of miPALM using the DIP network as input. **B)** Precision and recall of the two versions of miPALM. Circles, F-measures measured against CYC08 (C) or YHTP08 (Y). Lines, F-measure contours.

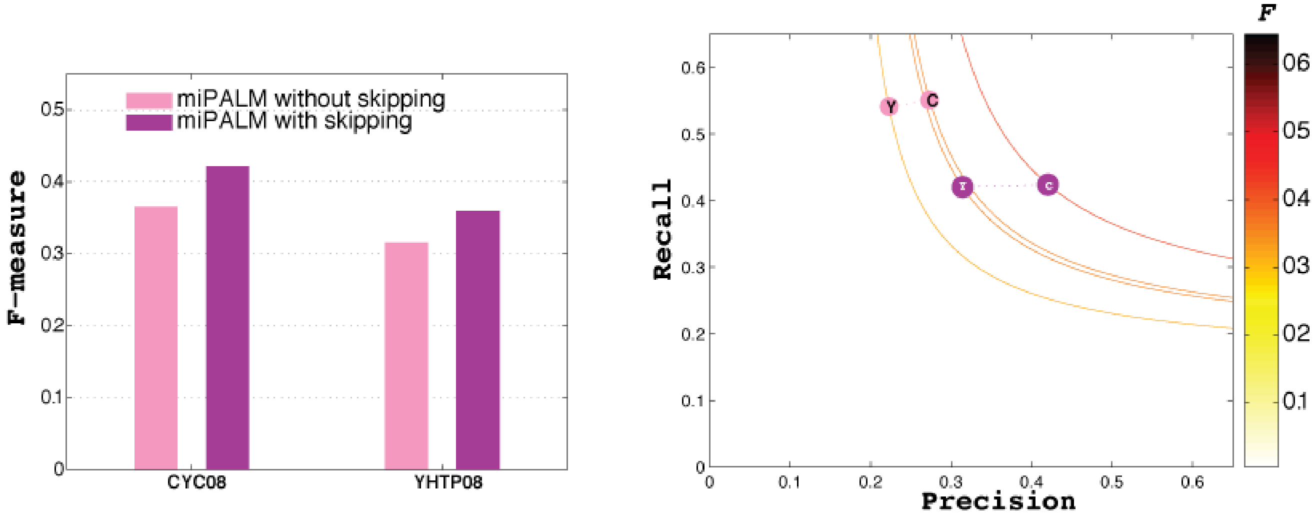

Figure S3. A small sub-network illustrating the relationship between S and $S^{'}$,where$S' = S \cup \{v^*\}$.After inclusion of the best node v^* into S,the direct neighbors N_s of S($N_s \cap S = \varnothing$) and $N_{s'}$ of S' ($N_{s'} \cap S' = \varnothing$)are related by the set operation,

$N_{s'} = (N_s - \{v^*\}) \cup (N_{v^*} - (N_s \cup S))$.

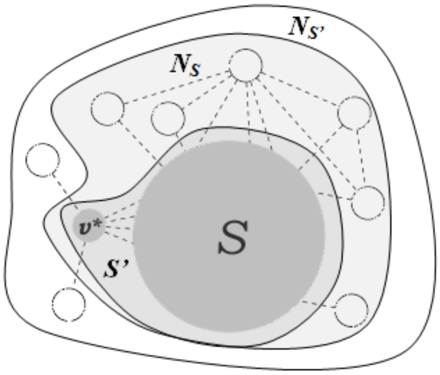

Figure S4. Optimal parameter search for miPALM.F-measure is plotted as a function of two variables *α* and *δ*. The global maximum F-measureswere found at *α*=0.364 and *δ*=2.4for the CYC08 set (**A**) and *α*=0.374 and *δ*=2.33 for the YHTP08 set (**B**). Yeast DIP full PPI network was used as the input.

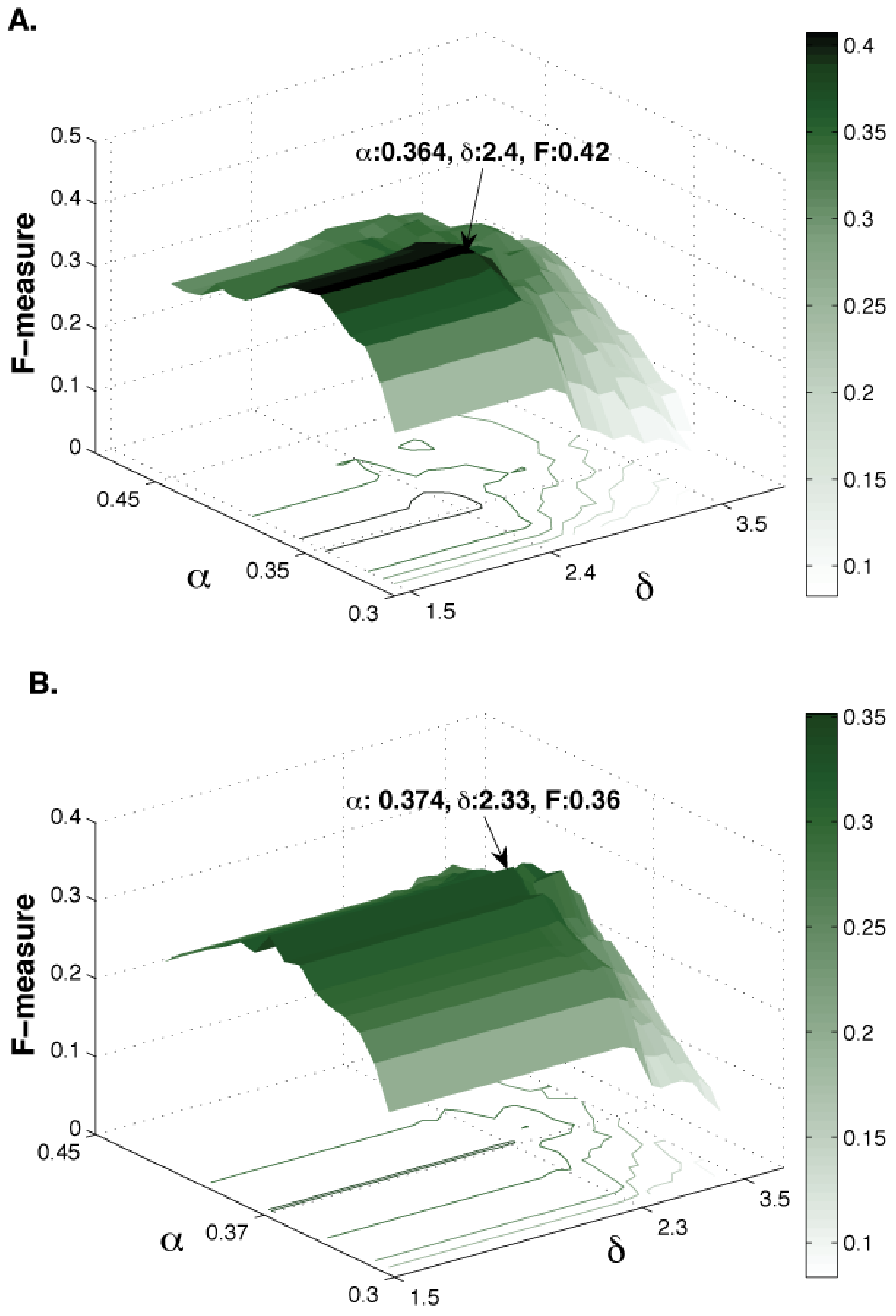

Figure S5. Overlap between miPALM predicted complexes using two different *alpha* values 0.364 and 0.374 for a given PPI network. Two input PPI networks were used: BioGRID and HC.

A) Overlap between protein members in the predicted complexes. **B)** Overlap between complexes. **C)** Overlap between novel complexes.

Result using BioGRID PPI network as input

A **B** **C**

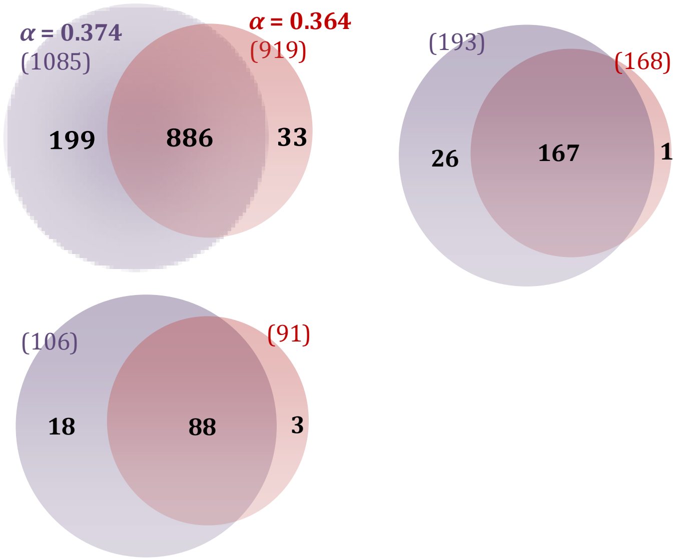

Result using HC PPI network as input

A **B** **C**

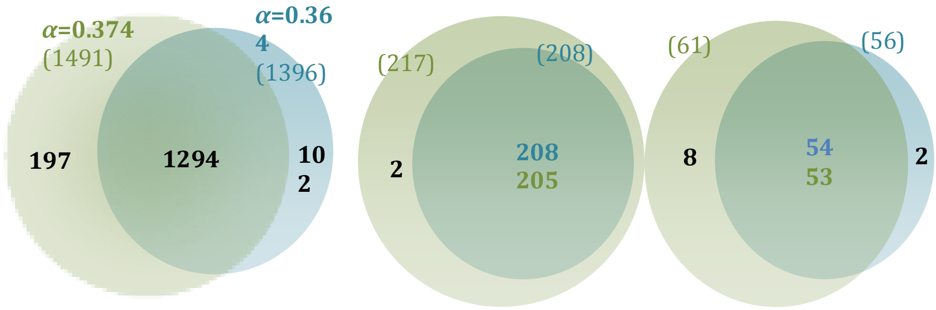

Figure S6. Overlap between miPALM predicted complexes using three PPI networks as inputs and miPALM alpha parameter of 0.364.

A) Overlap between protein members in the predicted complexes. **B)** Overlap between complexes. **C)** Overlap between novel complexes. Yellow, predicted complexes using DIP network; Blue, predicted complexes using HC PPI network; Red, predicted complexes using BioGRID PPI network.

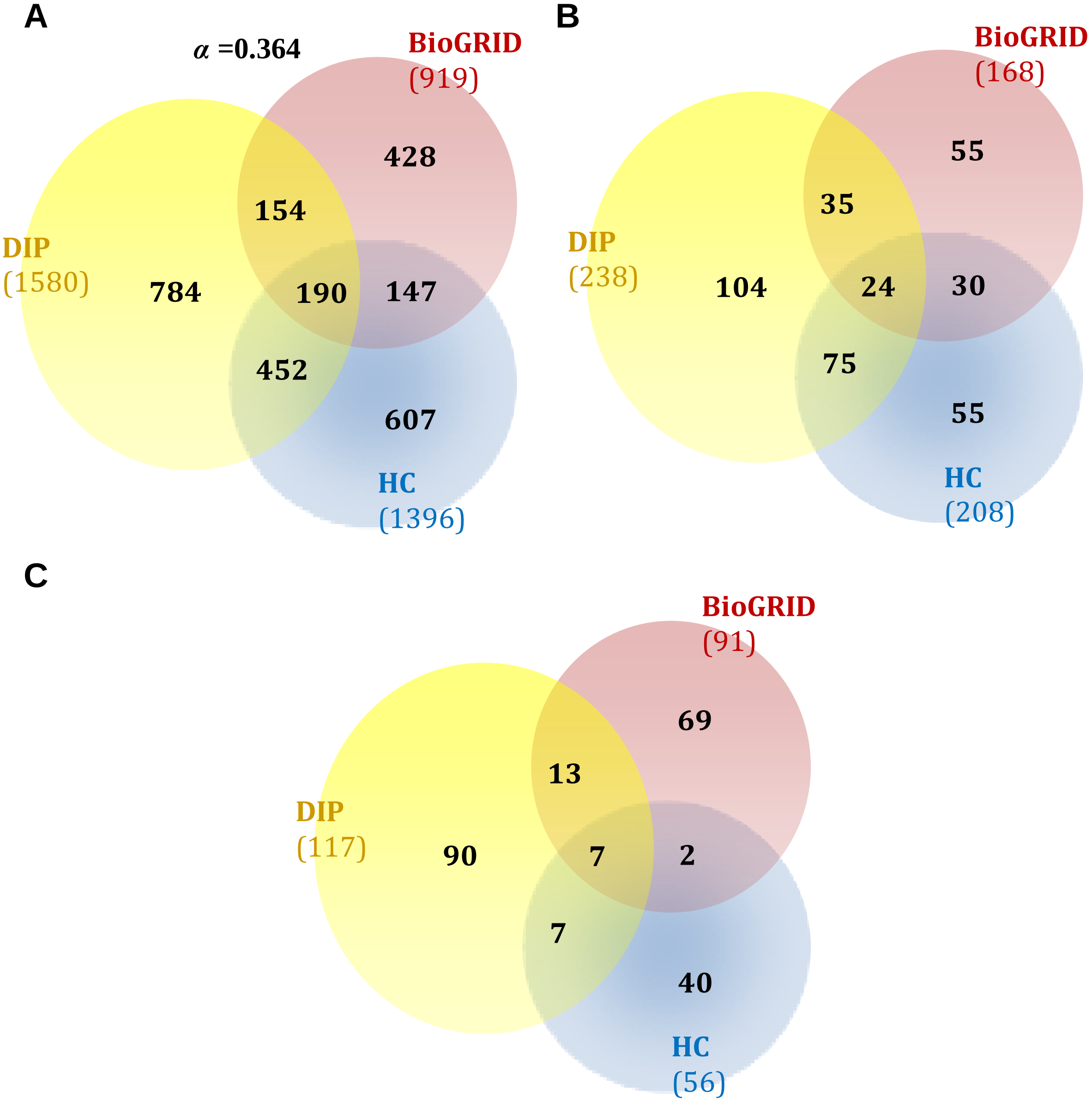

Figure S7. Overlap between miPALM predicted complexes using three PPI networks as inputs and miPALM alpha parameter of 0.374.

A) Overlap between protein members in the predicted complexes. **B)** Overlap between complexes. **C)** Overlap between novel complexes. Yellow, predicted complexes using DIP network; Purple, predicted complexes using HC PPI network; Green, predicted complexes using BioGrid PPI network.

A

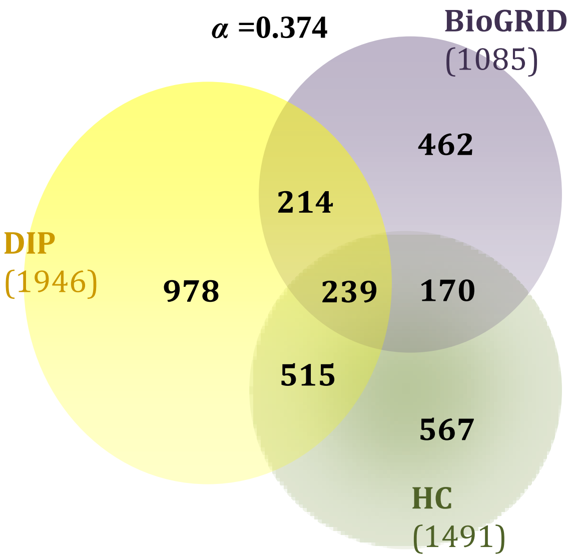

B

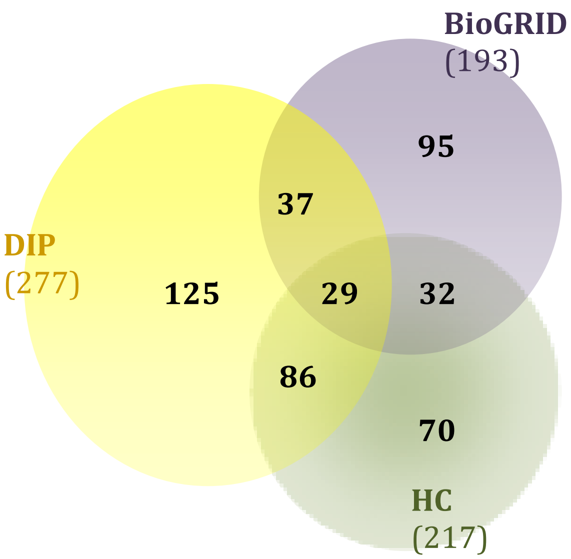

C

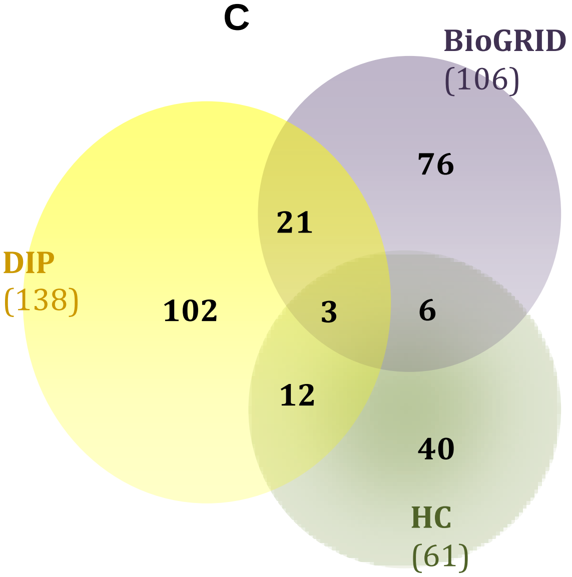

Figure S8. Performance of five complex-finding methods using the separation measure proposed by Brohee and van Helden. Brown, cluster-wise separation. A cluster-wise separation value of 1 indicates a predicted complex (cluster) fully and exclusively comprises all the members of one or several complexes, i.e. it contains all the proteins of the considered complex(es), and no other clusters contains any of these proteins. Yellow, complex-wise separation. It indicates how well a given gold standard complex is represented by one or more predicted clusters. A complex-wise separation value of 1 indicates all the proteins in a complex are contained in one or more clusters that do not contain any other protein. Y-axis, geometric mean separation (overall separation). The DIP full PPI network was used as the input.

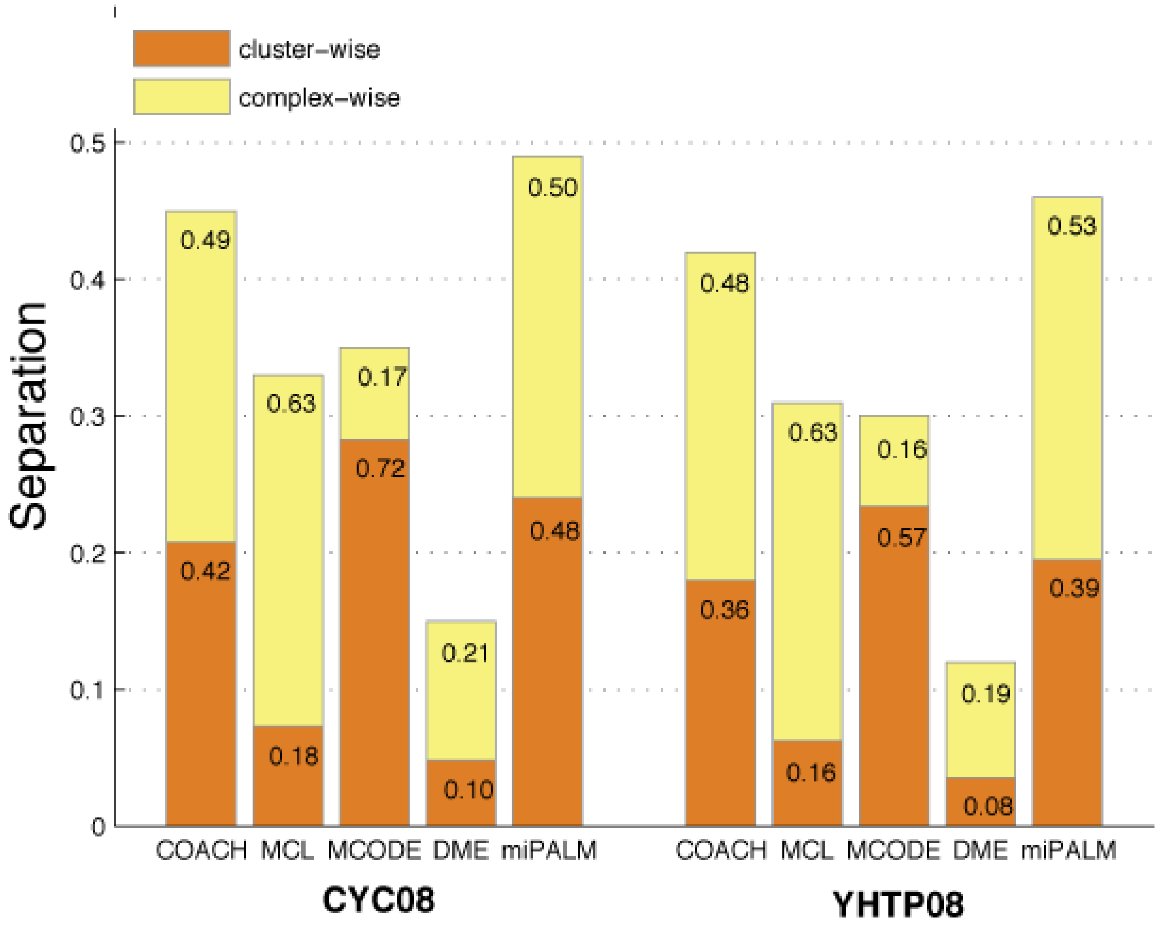

Figure S9. Predicted complexes that match known complexes at different overlapping thresholds. Shown are total number of matched complexes to the CYC08 (red) and YHTP08 sets of known complexes. For each overlapping threshold, precision (first percentage) and recall (second percentage) are also shown. The DIP full PPI network was used as the input.

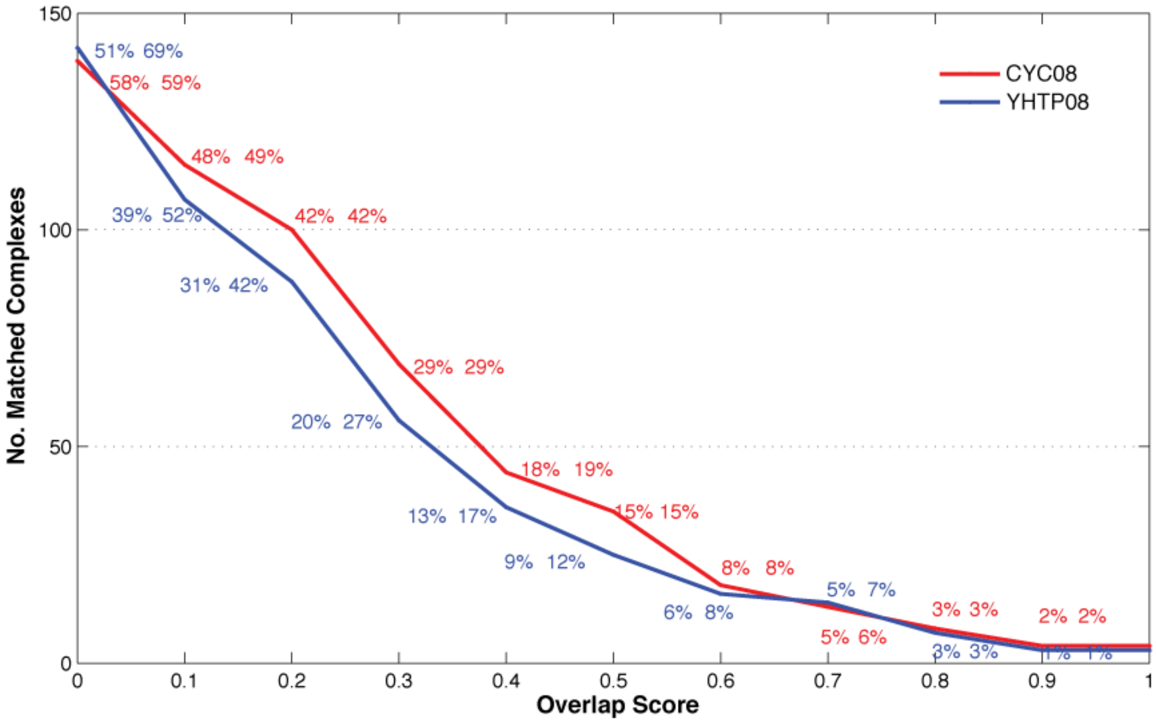

Table S4 - Performance of five algorithms with the best parameters optimized on CYC08 and YHTP08 sets and the DIP PPI network as the input. For each method, two sets of predictions were made based on the parameter optimized on CYC08 and YHTP08.

Algorithm	# Predicted Clusters		Performance Measure			
			Precision/Recall/F-measure		Separation	
	CYC08	YHTP08	CYC08	YHTP08	CYC08	YHTP08
COACH	271	271	0.42/0.48/0.45	0.35/0.46/0.40	0.45	0.42
MCODE	57	57	0.44/0.11/0.17	0.32/0.09/0.14	0.35	0.30
MCL	830	830	0.15/0.52/0.23	0.14/0.57/0.22	0.33	0.31
DME	487	503	0.09/0.19/0.12	0.08/0.19/0.11	0.15	0.12
miPALM	238	277	0.42/0.42/0.42	0.31/0.42/0.36	0.49	0.46

Table S5 - Performance of five algorithms with the best parameters optimized on CYC08 and YHTP08 sets and the BioGrid PPI network as input. For each method, two sets of predictions were made based on the parameter optimized on CYC08 and YHTP08. *, DME predicted a large number of complexes (>15,000) using the BioGrid input network, which are unlikely based on current knowledge about the number of protein complexes in *S. cerevisiae*. Therefore, its result is not reported here.

Algorithm	# Predicted Clusters		Performance Measure			
			Precision/Recall/F-measure		Separation	
	CYC08	YHTP08	CYC08	YHTP08	CYC08	YHTP08
COACH	642	642	0.34/0.58/0.43	0.31/0.51/0.38	0.25	0.19
MCODE	55	55	0.35/0.08/0.13	0.18/0.05/0.08	0.27	0.20
MCL	1620	1620	0.02/0.11/0.03	0.02/0.14/0.03	0.07	0.07
DME*	-	-	-	-	-	-
miPALM	168	193	0.39/0.26/0.31	0.25/0.23/0.24	0.44	0.39

Table S6 - Performance of five algorithms with the best parameters optimized on CYC08 and YHTP08 sets and the high-confidence BioGrid PPI network (HC network) as input. For each method, two sets of predictions were made based on the parameter optimized on CYC08 and YHTP08.

**Algorithm**	**# Predicted Clusters**		**Performance Measure**			
			**Precision/Recall/F-measure**		**Separation**	
	CYC08	YHTP08	CYC08	YHTP08	CYC08	YHTP08
COACH	274	274	0.62/0.64/0.63	0.56/0.59/0.58	0.49	0.48
MCODE	116	116	0.74/0.36/0.48	0.61/0.34/0.43	0.51	0.50
MCL	478	478	0.10/0.17/0.12	0.07/0.15/0.10	0.19	0.16
DME	2892	3136	0.30/0.86/0.44	0.31/0.84/0.45	0.18	0.12
miPALM	208	215	0.58/0.47/0.52	0.51/0.51/0.51	0.52	0.52
